# Associations of Adiponectin with Adiposity, Insulin Sensitivity, and Diet in Young, Healthy, Mexican Americans and Non-Latino White Adults

**DOI:** 10.3390/ijerph13010054

**Published:** 2015-12-22

**Authors:** Rocio I. Pereira, Cecilia C. Low Wang, Pamela Wolfe, Edward P. Havranek, Carlin S. Long, Daniel H. Bessesen

**Affiliations:** 1 Anschutz Medical Campus, University of Colorado, Aurora, CO 80045, USA; cecilia.lowwang@ucdenver.edu (C.C.L.W.); pamela.wolfe@ucdenver.edu (P.W.); daniel.bessesen@ucdenver.edu (D.H.B.); 2 Denver Health Medical Center, Denver, CO 80204, USA; ed.havranek@dhha.org (E.P.H.); carlin.long@dhha.org (C.S.L.)

**Keywords:** adiponectin, insulin resistance, diet, Mexican American

## Abstract

Low circulating adiponectin levels may contribute to higher diabetes risk among Mexican Americans (MA) compared to non-Latino whites (NLW). Our objective was to determine if among young healthy adult MAs have lower adiponectin than NLWs, independent of differences in adiposity. In addition, we explored associations between adiponectin and diet. This was an observational, cross-sectional study of healthy MA and NLW adults living in Colorado (U.S.A.). We measured plasma total adiponectin, adiposity (BMI, and visceral adipose tissue), insulin sensitivity (IVGTT), and self-reported dietary intake in 43 MA and NLW adults. Mean adiponectin levels were 40% lower among MA than NLW (5.8 ± 3.3 *vs.* 10.7 ± 4.2 µg/mL, *p* = 0.0003), and this difference persisted after controlling for age, sex, BMI, and visceral adiposity. Lower adiponectin in MA was associated with lower insulin sensitivity (R^2^ = 0.42, *p* < 0.01). Lower adiponectin was also associated with higher dietary glycemic index, lower intake of vegetables, higher intake of trans fat, and higher intake of grains. Our findings confirm that ethnic differences in adiponectin reflect differences in insulin sensitivity, but suggest that these are not due to differences in adiposity. Observed associations between adiponectin and diet support the need for future studies exploring the regulation of adiponectin by diet and other environmental factors.

## 1. Introduction

Adiponectin is an insulin-sensitizing hormone produced and released into the circulation primarily by adipose tissue [1]. Low adiponectin levels are associated with increased risk of type 2 diabetes in a dose-dependent relationship, across diverse populations, including Hispanics [2,3,4]. In a sub-analysis of the Diabetes Prevention Program (DPP) study, a ~3 µg/mL lower baseline adiponectin level in individuals with pre-diabetes corresponded to a 20%–40% higher rate of progression to diabetes [3]. Adiponectin levels vary by ethnicity and have been reported to be lower among Hispanics than non-Latino whites [3,5], raising the possibility that ethnic differences in adiponectin levels contribute to differences in diabetes risk. 

Several gaps in knowledge related to adiponectin in Hispanics still remain. First, previous studies have compared adiponectin levels in non-Latino whites to those in Hispanics as a group, rather than studying individuals from one single Hispanic-origin group. Since the term “Hispanic” includes individuals from many different geographical areas, with varied genetic backgrounds, and different cultural practices (language, diet, *etc.*), one would expect that biological factors differ as well. In fact, in the United States, prevalence of diabetes is much higher among Mexican Americans (~19%) than among South Americans (~10%) or Cubans (13%–14%) [6]. Assuming similar differences in adiponectin levels across Hispanic origin groups, the difference between adiponectin levels in Mexican Americans compared to non-Latino whites would be expected to be higher than that between a heterogeneous group of Hispanics and non-Latino whites. Second, although previous studies have consistently reported lower adiponectin levels among Hispanics, and some have controlled for age, sex, BMI, and waist circumference [3,5,7], none to our knowledge, has controlled for visceral adiposity as measured by gold-standard CT. Therefore, whether lower adiponectin levels among Hispanics are explained by higher visceral adiposity remains unknown. Third, only two studies (our previous study [7] and the MESA study [5]) compared the association between adiponectin and insulin sensitivity/resistance, and both of these used a surrogate measure (HOMA-IR) to estimate insulin resistance. Finally, recent data suggest associations between diet components and adiponectin, and an effect of dietary changes on adiponectin levels in mostly white European populations [8,9]. In particular, high fiber intake [10,11] and low glycemic load diets [12] increase adiponectin concentrations. However, these associations have not been explored in ethnic-minority populations. 

The aim of the present study was to test the hypothesis that adiponectin concentrations are lower in healthy Mexican Americans compared to non-Latino white adults, controlling for visceral adiposity, and that lower adiponectin in Mexican Americans is associated with lower insulin sensitivity. In addition, we explored the associations of adiponectin with diet components.

## 2. Methods

### 2.1. Study Population

We recruited Mexican American and non-Latino white men and women, 18–60 years of age, with BMI between 20 and 35 kg/m^2^ and no evidence of diabetes as determined by a medical history and screening fasting glucose <126 mg/dL. Eligible Mexican American participants reported having at least three grandparents who were born in Mexico and non-Latino white participants denied having any grandparents of Latino descent. Individuals with diabetes or other significant medical conditions such as heart, kidney, or liver failure were excluded. Women who were pregnant, planning to become pregnant during the course of the study, or breast feeding were also excluded. The study protocol was approved by the Colorado Multiple Institutional Review Board and all study participants provided written informed consent to participate.

### 2.2. Study Design 

We conducted an observational, cross-sectional study. Recruitment of participants was done through interest meetings and fliers at schools and community centers in Aurora, Colorado, and through emails and fliers at the University of Colorado Anschutz Medical Campus. Interested individuals were screened by phone, and then invited to attend a screening visit during which the study protocol was reviewed and informed consent was obtained. At the screening visit, potential participants had a medical history, physical exam, and blood tests to rule out significant medical illness. Eligible individuals returned for a second study visit where they completed the Baecke physical activity questionnaire [13] and the Block food frequency questionnaire (NutritionQuest, Berkeley, CA, USA), and had a DXA scan (Hologic Discovery, Hologic, Inc., Bedford, MA, USA). Participants were then asked to wear a pedometer and log their daily steps for 7 days. Participants attended a final study visit during which they had a fasting blood draw for measurement of adiponectin, insulin, and glucose, a frequently-sampled IVGTT [14] for assessment of insulin sensitivity measures, and a two-slice CT scan at L2/L3 and L4/L5 for measurement of visceral adipose tissue (VAT). 

### 2.3. Measurements and Definitions 

Laboratory measures were performed by the Colorado Clinical and Translational Sciences Institute (CCTSI) Core Laboratory. Blood samples were collected in EDTA-containing tubes. Samples were transported on ice and immediately centrifuged at 20 °C at 3000 rev/min for 15 min. The supernatants were stored in aliquots at −80 °C. Adiponectin and insulin plasma concentrations were measured by RIA (Millipore, Billerica, MA, USA). Intra- and inter-assay precisions for the adiponectin assay are 1.78%–3.59% CV and 6.90%–9.25% CV respectively. HOMA2-IR was calculated from fasting insulin and glucose using the HOMA2 calculator program (University of Oxford, Oxford, UK, 2004); BMI was calculated as kg/m^2^; acute insulin response (AIR) and insulin sensitivity (S_i_) were calculated using the Bergman MINMOD program [14] with glucose and insulin values obtained during a frequently-sampled IVGTT. Daily vitamin D intake included intake from both food sources and dietary supplement intake as reported in the food frequency questionnaire.

### 2.4. Statistical Analyses 

Unadjusted differences in all variables between ethnic groups and gender were evaluated using two-group *t* tests. The primary outcome, difference in adiponectin concentrations between ethnic groups, was further tested using an ANCOVA model controlling for age, gender, BMI, and visceral adiposity. Additional covariates were added based on clinical relevance or correlation with the outcome measure where the *p* value was < 0.01. Association between adiponectin and insulin sensitivity by ethnicity was evaluated in the same manner. Insulin sensitivity was log transformed for analysis. Associations between adiponectin and dietary data for the entire cohort were evaluated using Spearman’s correlation coefficient. SAS version 9.2 (SAS Institute Inc., Cary, NC, USA) was used for all statistical analyses.

## 3. Results

A total of 43 individuals were studied. Data for one individual with undetectable circulating adiponectin (attributed to sample degradation) was excluded from the analyses. Demographic characteristics and clinical measures of study participants stratified by ethnicity are presented in Table 1. 

Mexican Americans were shorter and had higher BMI, waist circumference, and VAT than non-Latino whites. Mexican Americans also had higher AIR, lower S_i_, and lower adiponectin. As expected, men in both ethnic groups had lower adiponectin than women, but gender differences were less pronounced among Mexican Americans. Mexican American men and women had lower adiponectin than their non-Latino white counterparts (4.3 ± 1.8 µg/mL *vs.* 7.7 ± 4.2 µg/mL, *p* = 0.06 in men, 6.7 ± 3.8 µg/mL *vs.* 12.1 ± 3.7 µg/mL, *p* = 0.001 in women) though this difference did not reach statistical significance in the men. 

**Figure 1:**
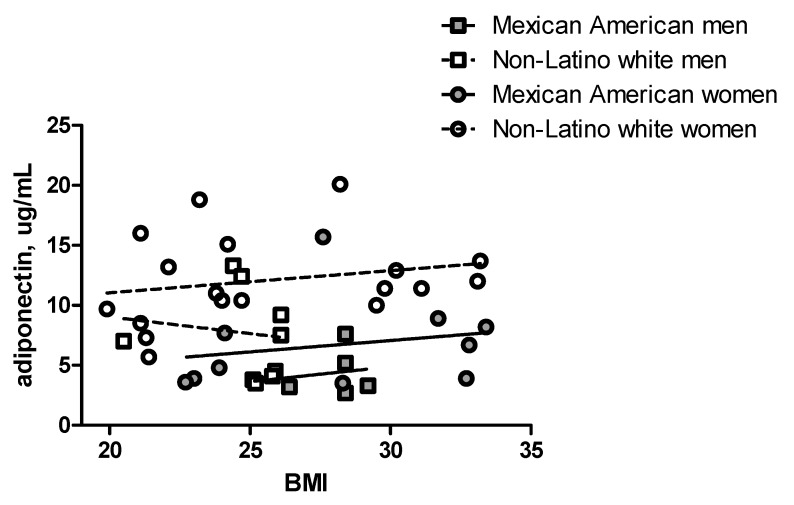
Differences in adiponectin concentrations between Mexican Americans and non-Latino Whites across BMI Ranges. Symbols represent adiponectin concentrations and BMI for Mexican American women (●), men (■), and non-Latino white women (○), and men (□). Lines represent linear regression lines for Mexican Americans (solid line) and non-Latino whites (dashed lines) with higher/longer lines representing women of each group. Differences in intercepts: *p* < 0.001 for women, *p* = 0.20 for men).

On multivariate analysis (Table 2), Mexican American ethnicity was associated with ~40% lower mean adiponectin concentrations (estimate (95% CI): −4.2 (−6.7, −1.8) µg/mL) after controlling for age, gender, BMI, and visceral adiposity. At each BMI level, Mexican American men and women had lower mean adiponectin compared to their non-Latino white counterparts (Figure 1).

Mexican Americans with low adiponectin had low insulin sensitivity (R^2^ = 0.42, *p* < 0.01), but this association was not observed among non-Latino whites. In contrast to the association between adiponectin and insulin sensitivity among Mexican Americans, the association between BMI and insulin sensitivity was not statistically significant among Mexican Americans, and weak (R^2^ = 0.20, *p* < 0.05) among non-Latino whites.

**Table 1:** Demographic characteristics and clinical measures by ethnicity.

Variable	Combined (*n* = 42)	Mexican American (*n* = 16)	Non-Latino White (*n* = 26)	*p*
Age (years)	34.5 ± 9.1	35.0 ± 6.3	34.2 ± 10.5	0.77
Sex (women/men)	28/14	10/6	18/8	0.66
Height (cm)	166.7 ± 11.0	161.7 ± 9.4	169.8 ± 11.0	0.02
Weight (kg)	73.3 ± 11.8	73.0 ± 10.2	73.5 ± 12.9	0.88
BMI (kg/m^2^)	26.4 ± 3.9	27.9 ± 3.5	25.5 ± 3.9	0.04
Waist circumference * (cm)	85.6 ± 9.3	89.4 ± 8.5	83.2 ± 9.0	0.03
SA * (cm^2^)	288 ± 110	319 ± 89	271 ± 117	0.19
VAT * (cm^2^)	71 ± 45	92 ± 48	60 ± 40	0.03
VAT/SAT	0.25 ± 0.15	0.30 ± 0.17	0.23 ± 0.14	0.17
Lean mass * (kg)	48.6 ± 10.2	47.4 ± 9.8	49.3 ± 10.5	0.57
Fasting glucose (mmol/L)	5.0 ± 0.4	5.1 ± 0.3	4.9 ± 0.4	0.02
Fasting insulin (pmol/L)	94.5 ± 61.8	120.1 ± 77.8	78.5 ± 43.8	0.03
HOMA2-IR	2.0 ± 1.2	2.5 ± 1.5	1.7 ± 0.9	0.03
AIR (µU/mL × 10 min)	667 ± 444	861 ± 497	548 ± 369	0.02
S_i_ (×10^−4^/min/µU/mL)	3.8 ± 2.1	2.7 ± 1.7	4.6 ± 1.9	0.002
DI (×10^−4^/min)	2174 ± 1674	2078 ± 2201	2233 ± 1296	0.78
CRP (mg/L)	1.6 ± 1.7	2.2 ± 2.4	1.2 ± 0.9	0.09
Leptin (ng/mL)	13.3 ± 10.6	15.2 ± 11.2	12.2 ± 10.2	0.4
Adiponectin (µg/mL)	8.9 ± 4.5	5.8 ± 3.3	10.7 ± 4.2	0.0003
LAR (ng/µg)	1.8 ± 1.6	2.8 ± 2.0	1.3 ± 0.9	0.001

Notes: Mean ± SD; AIR= Acute Insulin Response; DI, Disposition Index; LAR = leptin-adiponectin ratio; SAT = subcutaneous adipose tissue; S_i_ = Insulin Sensitivity; VAT = visceral adipose tissue * Up to 2 cases missing.

**Table 2:** ANCOVA with adiponectin as the dependent variable.

	Model 1	Model 2
Variable	EST (95% CI)	EST (95% CI)
Mexican American ethnicity	−4.6 (−7.2, −2.0)	−4.2 (−6.7, −1.8)
Male gender		−2.2 (−0.4, −4.7)
Age		0.2 (0.03, 0.3)
BMI		0.5 (0.1, 1.0)
VAT		−0.1 (−0.1, −0.01)
R^2^	0.23	0.54

Self-reported dietary intake data obtained via food-frequency questionnaire was compared for the two ethnic groups (Table 3; reported per 1000 kilocalories (kcal)). There were no ethnic differences in daily caloric intake or in major macronutrient distributions (carbohydrates, protein, and fat). However, ethnic differences were observed in macronutrient components. Mexican Americans reported higher servings of grains (including whole grains), but lower servings of fruits and vegetables compared to non-Latino whites. Similarly, Mexican Americans reported higher intake of bean fiber and lower intake of fruit and vegetable fiber. Mexican Americans also reported higher intake of trans fats, but intake of saturated fat was similar in the two groups. Intakes of dairy, total fiber, glycemic index (GI), and glycemic load (GL), were also similar between the two ethnic groups.

**Table 3:** Daily dietary measures by ethnicity and gender.

	Mexican American (*n* = 16)	Non-Latino White (*n* = 26)	*p* ^1^
Energy (kcal)	1960 ± 790	1734 ± 621	0.31
Carbohydrate (g/1000 kcal)	131.0 ± 18.2	122.0 ± 18.3	0.13
Protein (g/1000 kcal)	39.2 ± 8.1	38.0 ± 6.6	0.60
Fat (g/1000 kcal)	36.4 ± 5.4	37.7 ± 6.0	0.49
Grain servings (per 1000 kcal)	3.3 ± 1.1	2.6 ± 0.8	0.01
Whole grain servings (per 1000 kcal)	1.0 ± 0.8	0.4 ± 0.4	0.001
Fruit servings (per 1000 kcal)	0.5 ± 0.5	1.0 ± 0.6	0.02
Vegetable servings (per 1000 kcal)	1.0 ± 0.6	1.7 ± 1.1	0.03
Meat servings (per 1000 kcal)	1.6 ± 0.5	1.1 ± 0.3	0.001
Dairy servings (per 1000 Kcal)	0.8 ± 0.5	0.8 ± 0.5	0.66
Fat servings (per 1000 kcal)	2.0 ± 0.9	1.7 ± 0.7	0.23
Fiber (g/1000 kcal)	9.8 ± 2.9	10.4 ± 3.5	0.57
Bean fiber (g/1000 kcal)	2.2 ± 1.5	1.3 ± 0.7	0.008
Fruit/veg. fiber (g/1000 kcal)	2.4 ± 1.2	4.7 ± 2.8	0.005
Grain fiber (g/1000 kcal)	4.7 ± 1.6	4.3 ± 2.6	0.53
GI, glucose	49.9 ± 2.1	48.8 ± 3.2	0.23
GL, glucose (per 1000 kcal)	60.6 ± 9.8	54.7 ± 10.8	0.83
Saturated fat (g/1000 kcal)	11.4 ± 2.3	11.6 ± 2.8	0.81
Trans fat (g/1000 kcal)	1.3 ± 0.4	1.1 ± 0.3	0.03
Vitamin D (IU/1000 kcal)	147 ± 93	198 ± 157	0.24
Omega 3 FA (g/1000 kcal)	0.7 ± 0.2	0.9 ± 0.3	0.16

Note: ^1^ Based on a two-group *t* test for ethnic groups pooling across gender.

In univariate analyses of dietary data for the entire cohort, several dietary factors were associated with higher adiponectin including higher fruit and vegetable fiber intake (*r* = 0.50, *p* = 0.0007), lower glycemic index *r* = −0.47, *p* = 0.0017), fewer grain servings (*r* = −0.42, *p* = 0.005), and lower trans fat intake (*r* = −0.4, *p* = 0.008).

**Figure 2:**
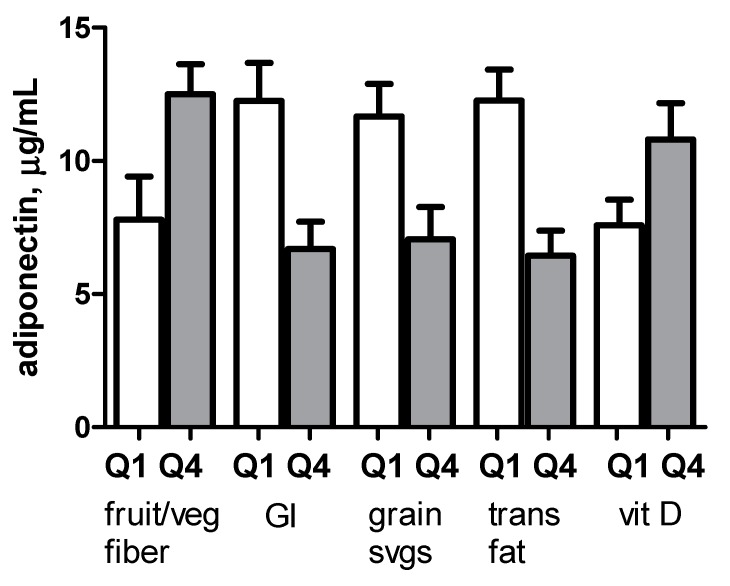
Adiponectin concentrations among individuals in lowest and highest gender-specific quartile for each dietary component. Mean and SEM of adiponectin concentrations for individuals in the lowest (Q1, white bar) and highest (Q4, shaded bar) gender-specific quartile for intake of each dietary factor. GI, glycemic index.

Compared to individuals in the lowest gender-specific quartiles for fruit/vegetable fiber, individuals in the highest quartiles had adiponectin concentrations that were 4.7 µg/mL higher, *p* = 0.03. Conversely, compared to individuals in the lowest gender-specific quartiles for glycemic index, grain servings, and trans fat intake, individuals in the highest quartiles had adiponectin concentrations that were 5.6 µg/mL, 4.6 µg/mL, and 5.8 µg/mL lower (*p* = 0.005, *p* = 0.01 and *p* = 0.001) respectively (Figure 2). On multivariate analyses including ethnicity, gender, BMI, and each individual dietary factor, dietary glycemic index, grain servings, and vitamin D remained independently associated with adiponectin levels (*p*-values 0.01, 0.01, and 0.007 respectively), while associations with fruit/vegetable fiber and trans fat intake did not reach statistical significance (*p*-values 0.1 and 0.15). 

## 4. Discussion

Our findings confirm previous observations of lower adiponectin levels in Mexican Americans compared to non-Latino whites, and verify that this difference remains after controlling for visceral adiposity. Furthermore, our finding of a stronger association between adiponectin and insulin sensitivity among young healthy Mexican Americans compared to non-Latino whites suggests that adiponectin may be a stronger contributor to diabetes risk in the former population. Finally, our findings of associations between adiponectin and dietary components suggest a need for future research to explore the effects of dietary components on adiponectin, insulin sensitivity, and diabetes risk in Mexican Americans. 

Interpretation of our findings is limited by the observational nature of the study which prevents us from establishing causation in the observed associations. The relatively small sample size precluded a more thorough analysis of all the mechanisms involved in adiponectin regulation. Specifically, a larger sample size would be needed to definitively establish that dietary intake is associated with adiponectin after controlling for all potential confounders. We measured multiple associations between adiponectin and dietary components, and therefore some of associations having significant *p* values may have occurred by chance. We did not measure the expression of adiponectin receptors which likely also contribute to adiponectin’s insulin-sensitizing actions and may vary by ethnicity. Lastly, we did not measure concentrations of high-molecular weight adiponectin which is believed to be the active form of the molecule. Though a study of genetics effects on adiponectin regulation was beyond the scope of this work, we attempted to limit genetic variability by limiting our focus on Mexican Americans rather than a more heterogeneous Hispanic population. Another strength of the study is accurate measures of visceral adiposity by CT rather than using waist circumference as a surrogate marker, and of insulin sensitivity by frequently-sampled IVGTT rather than estimating insulin sensitivity/resistance by HOMA alone.

Adiponectin concentrations are known to vary by ethnicity, and lower mean adiponectin concentrations have previously been reported in different ethnic/racial subgroups including Hispanics. Adiponectin was lower in ethnic/racial minorities, including Hispanics, in the Diabetes Prevention Program study after adjustment for age, BMI, and waist circumference [3]. Our findings are also consistent with those of the MESA study [5] in which adiponectin concentrations were lower in Hispanic compared to non-Latino white subjects after controlling for BMI. However, neither of these studies controlled for ethnic differences in visceral adiposity. Observed ethnic/racial differences in adiponectin concentrations in these two studies are more modest than those observed in our present study. However, the Hispanic groups in both of these studies were not limited to Mexican Americans and included other Hispanic-origin groups with lower risk of diabetes (*i.e*., South Americans and Cubans) that would be expected to have higher adiponectin concentrations than Mexican Americans. We have previously reported that among patients with hypertension and other cardiovascular disease risk factors, Mexican Americans have lower adiponectin concentrations than non-Latino whites, after controlling for BMI and waist circumference [7]. Our present report expands our observations to young, healthy, community-dwelling individuals, and contributes to the existing evidence that ethnic differences in adiponectin are not due to differences in adiposity measures including visceral adiposity. 

Adiponectin is believed to improve insulin sensitivity through a number of different mechanisms. In addition, adiponectin has potent anti-apoptotic effects which are believed to prevent lipid-induced pancreatic beta cell apoptosis [15]. Both of these actions would be expected to be protective against diabetes. Consistent with this evidence, lower adiponectin is associated with incident diabetes across diverse populations [2,4]. However, it has also been suggested that adiponectin concentrations are themselves regulated by insulin and that low adiponectin observed in settings of low insulin sensitivity are due to compensatory hyperinsulinemia [16]. Thus, the directionality of the association between adiponectin and insulin sensitivity remains in question. Ours is a correlational study and does not shed additional light on this relationship. However, our findings support the utility of adiponectin over adiposity measures as an indicator of insulin sensitivity and diabetes risk among Mexican Americans. 

Multiple recent studies have examined associations between dietary factors and adiponectin and the effects of dietary modifications on adiponectin concentrations [8,9,17]. To our knowledge, ours is the first to examine associations between dietary factors and adiponectin among Mexican Americans. Our findings of lower adiponectin in association with lower intake of fruit and vegetable fiber, higher dietary glycemic index, and higher intakes of grains and trans fat, are consistent with previous observations among non-Latino whites [18,19]. In the Nurses’ Health Study of women with type 2 diabetes [20] and in the Health Professionals’ Follow-up Study of men with type 2 diabetes [18], adiponectin was lower among individuals reporting diets high in glycemic index and glycemic load, and higher among individuals reporting high cereal and fruit fiber intake. In another study [21], adiponectin concentrations decreased from baseline after consumption of a high fat meal in non-diabetic subjects and in patients with type 2 diabetes. In patients with diabetes, adiponectin concentrations also decreased after consumption of a high carbohydrate, low-fiber meal, but not after consumption of a high carbohydrate, high-fiber meal. Our finding of lower adiponectin with higher whole grain intake was unexpected and is counterintuitive. This observation may reflect an effect of the specific whole grain foods consumed by the individuals studied, or other related dietary components that we did not measure. Our findings suggest that dietary modifications may improve adiponectin concentrations and highlight the need for future more definitive studies in this research area. 

While it appears clear that adiponectin is decreased in Mexican Americans, the molecular and environmental regulators involved in this ethnic difference are unknown. The question of whether adiponectin concentrations can be increased through dietary modification in Mexican Americans and other populations with low adiponectin remains unanswered. Furthermore, whether or not such increases in adiponectin would be associated with improved insulin sensitivity and decreased risk for diabetes is unknown. Future research to address these questions is warranted. 

## 5. Conclusions

In conclusion, the present study showed that Mexican Americans have lower circulating adiponectin levels than non-Latino whites, independent of differences in adiposity. Lower adiponectin among Mexican Americans was associated with lower insulin sensitivity which is a known risk factor for diabetes. Associations between dietary intake and adiponectin where observed, suggesting that dietary modifications may be a potential tool for improved insulin sensitivity and decreased risk for diabetes among Mexican Americans.

## List of Abbreviations

AIR: acute insulin response
CCTSI: Colorado Clinical and Translational Sciences Institute
CRP: C-reactive protein
CT: computed tomography
DI: disposition index
DXA: dual-energy X-ray absorptiometry
FA: fatty acids
GI: glycemic index
GL: glycemic load
IU: international units
LAR: leptin-adiponectin ratio
DXA: dual-energy X-ray absorptiometry
FA: fatty acids
GI: glycemic index
GL: glycemic load
IU: international units
LAR: leptin-adiponectin ratio
Q: quartile
R^2^: r squared
SAT: subcutaneous adipose tissue
S_i_: insulin sensitivity
VAT: visceral adipose tissue
